# Supplementary material for: SC-GROG followed by L+S reconstruction with multiple sparsity constraints for accelerated Golden-angle-radial DCE-MRI
Source: PLoS One. 2025 Feb 14;20(2):e0318102. doi: 10.1371/journal.pone.0318102 (PMC11828349; doi:10.1371/journal.pone.0318102)
Supplement: Attachment — Submitted filename: PLOS ONE Review.docx [file pone.0318102.s001.docx]

Major Comment

1. How MFISTA-VA is computationally better than the soft thresholding used in 1, 2, 6 and 9?
2. Can you explain the difference between this article? Shahzadi, Iram, et al. "Golden-angle radial sparse parallel MR image reconstruction using SC-GROG followed by iterative soft thresholding." *Applied Magnetic Resonance* 50 (2019): 977-988.
3. It seems your group has done quite a lot of work on GROG, GRASP and Compresses Sensing based reconstructions, it will be better if you can provide a comparison of results with this article as well.
4. Section 2.2 contains a lot of information from the original paper by Ricardo Otazo about L+S Decomposition Model. It is better to reduce this section to show how MFISTA-VA is applied for L+S Model.
5. Which algorithm was used to estimate the sensitivity maps?
6. Expression 3.2 there is no explanation how TemporalTV and and Temporal FFT is applied on sparse data separately.
7. “Table 4 shows that the peak DCE signal was degraded by 8.5% and 6.5% ….. ” Degraded in comparison of what ? Did you use any reference image? How about adding a non-binned image for comparison ?
8. In discussion section there is no mention of the reason why this SC GROG based method performs better than conventional methods. Which is very important for this section.

Minor Comments

1. Introduction: Paragraph 1: “physiological movement (e.g., respiratory, and cardiac motion) introduces blurring artefacts”, these movements don’t only add up to the blurring artifacts, there are more artifacts linked with these movements.
2. Introduction: Paragraph 3: “Temporal TV can suppress all the temporal variations”, how the spatial resolution is tackled?
3. Introduction: Paragraph 5: Repetitive sentence. “To generate good quality DCE MR images, intensity variation due to contrast agent and the respiratory motion must be separated from each other before image reconstruction”.
4. Theory Section: 2.1 Iterative Soft Thresholding Methods. It is good to talk about the thresholding methods, while on the other hand it is better to keep it precise to make it more readable. It is better to focus only on MFISTA-VA which is used in this paper.
5. “Tables 4 provides the peak and mean DCE signal intensity values in the three reconstruction schemes for Dataset-1 and Dataset-2 respectively”, are you comparing the complete image or just the sparse images ? How it behaves if you just compare sparse images?
6. Figure 4 is a bit confusing. It is not clear if you are talking about a 11 frames reconstruction, or 22 frames reconstruction in total or you are just showing results for these two particular frames.
7. There is not explanation of Figure 6 and Figure 7 in Results Section. And the plots are very blurry, and not suitable to be included in the paper in current form.
8. Figure 6 and 7, needs improvement, as they both need all the methods mentioned for comparison in the results section.

Formatting: There are quite a few grammatical errors, like

“NL-CG reconstruction which requires repeated NUFFT **ridding/degrading** to maintain”

“Where 𝑌𝑐 represents the under-sampled GROG-gridded Cartesian.”, complete this sentence.

The placement of figures and tables needs to be corrected. Like Figure 4, comes before table 4, while you talk about it later in the text. Same for the Tables with pseudo codes for algorithms.
