# Supplementary material for: SC-GROG followed by L+S reconstruction with multiple sparsity constraints for accelerated Golden-angle-radial DCE-MRI
Source: PLoS One. 2025 Feb 14;20(2):e0318102. doi: 10.1371/journal.pone.0318102 (PMC11828349; doi:10.1371/journal.pone.0318102)
Supplement: Attachment — Submitted filename: response to reviewers .docx [file pone.0318102.s002.docx]

# Response to Reviewers’ Comments

**(Manuscript Number:**  PONE-D-24-06314)

We are thankful to the reviewers for their deep and thorough review of our paper “SC-GROG followed by L+S reconstruction with multiple sparsity constraints for accelerated Golden-angle-radial DCE-MRI”. We have revised the manuscript in the light of their useful suggestions and comments. We hope that this revision has improved the paper to a level of their satisfaction. Number-wise answers to the specific comments/suggestions/queries are as follow:

| **Sr. No** | **Comments** | **Response** |
| --- | --- | --- |
|  | **Reviewer: 1** | |
| 1 | Abstract, row 5 from bottom  =======================  and (iii) reconstruction time.  =>  , (iii) reconstruction time, and (iv) image examples.  1. Introduction, row 11  =======================  In GRASP, data was  =>  In GRASP, data are  1. Introduction, row 12  =======================  contrast-enhanced images were reconstructed  =>  contrast-enhanced images are reconstructed  1. Introduction, row 26  =======================  Although GRASP based  =>  Although GRASP-based  1. Introduction, row 27  =======================  performance, however  =>  performance, however,  1. Introduction, row 29  =======================  NUFFT ridding/degrading  =>  NUFFT gridding/degrading  1. Introduction, row 12 from the bottom  ====================================  A point is missing.  1. Introduction, row 8 from the bottom  ================================  L+S  =>  L+S reconstruction  1. Introduction, row 5 from the bottom  ================================  ...work also uses SC-GROG ...  You have already mentioned that!  2.1 ITERATIVE SOFT THRESHOLDING ALGORITHMS, row 1  ================================  defined over complex  =>  defined over the complex  2.1 ITERATIVE SOFT THRESHOLDING ALGORITHMS, row 4  ===================================  Here h(x) represents sparsity  =>  Here h(x) represents the sparsity  2.1 ITERATIVE SOFT THRESHOLDING ALGORITHMS, row 5  ==================================  Please explain y!  2.1 ITERATIVE SOFT THRESHOLDING ALGORITHMS, row 5-6  ================================  Lipschitz constant, \|\| \|\| shows  =>  the Lipschitz constant, \|\| \|\| denotes  2.1 ITERATIVE SOFT THRESHOLDING ALGORITHMS, row 8-9  ================================  problem to reconstruct MR images, given the partially acquired k-space data d,  =>  problems, reconstruction of an MR image x,  2.1 ITERATIVE SOFT THRESHOLDING ALGORITHMS, row 11  ================================  In equation (2.2)  =>  In equation (2.2),  2.1 ITERATIVE SOFT THRESHOLDING ALGORITHMS, row 11  =================================  vector  =>  the vector  2.1 ITERATIVE SOFT THRESHOLDING ALGORITHMS, row 14  ================================  sparsity  =>  the sparsity  2.1 ITERATIVE SOFT THRESHOLDING ALGORITHMS, row 19  ================================  method Proximal-gradient  =>  methods, a Proximal-gradient  2.1 ITERATIVE SOFT THRESHOLDING ALGORITHMS, row 25  ================================  ... were proposed almost a decade ...  You repeat yourself!  2.1 ITERATIVE SOFT THRESHOLDING ALGORITHMS, row 17 from the bottom  =================================  major condition  =>  then a major condition  2.1 ITERATIVE SOFT THRESHOLDING ALGORITHMS, row 14 from the bottom  ====================================  here x* represents minimizer of the Psi, x_k denotes k_th iteration in M-FISTA with constant parameter W in  =>  Here x* represents the minimizer of Psi, x_k denotes the k_th iteration in M-FISTA with the constant parameter W as  2.1 ITERATIVE SOFT THRESHOLDING ALGORITHMS, row 13 from the bottom  =================================  satisfy convergence  =>  satisfy the convergence  2.2 MR RECONSTRUCTION USING ROBUST MATRIX DECOMPOSITION, row 2  ==================================  under sampled  =>  undersampled  2.2 MR RECONSTRUCTION USING ROBUST MATRIX DECOMPOSITION, row 3  =====================================  shows  =>  shows a  2.2 MR RECONSTRUCTION USING ROBUST MATRIX DECOMPOSITION, row 9  ===================================  less  =>  small  2.2 MR RECONSTRUCTION USING ROBUST MATRIX DECOMPOSITION, row 1 under TABLE 1  =================================  multi-coil under sampled dynamic MRI data, L+S decomposition  =>  multi-coil undersampled dynamic MRI data, the L+S decomposition  2.2 MR RECONSTRUCTION USING ROBUST MATRIX DECOMPOSITION, row 7 under TABLE 1  regularization parameters for L  =>  regularization parameters for the L  2.2 MR RECONSTRUCTION USING ROBUST MATRIX DECOMPOSITION, row 10 under TABLE 1  ====================================  ISTA/shrinkage operator  =>  The ISTA/shrinkage operator  2.2 MR RECONSTRUCTION USING ROBUST MATRIX DECOMPOSITION, row 24 under TABLE 1  ====================================  residual  =>  the residual  2.2 MR RECONSTRUCTION USING ROBUST MATRIX DECOMPOSITION, row 26 under TABLE 1  ====================================  proximal  =>  the proximal  2.2 MR RECONSTRUCTION USING ROBUST MATRIX DECOMPOSITION, row 29 under TABLE 1  =====================================  sum of nuclear  =>  the sum of the nuclear  3. PROPOSED METHOD, row 4  =========================  Flow-graph  =>  A flowchart  3. PROPOSED METHOD, row 5  =========================  image domain  =>  the image domain  Equation (3.1)  ==============  G.  =>  G  1 row below Equation (3.1)  ==========================  G shows GROG  =>  G is the GROG  2 rows below Equation (3.2)  ===========================  shows sparsity  =>  is the sparsity  3 rows below Equation (3.2)  ===========================  You write equation (2.7) but I think that you mean equation (2.8).  4. MATERIALS AND METHODS, row 3  ===============================  DCE-MRI data acquired from  =>  DCE-MRI dataset acquired from a  4. MATERIALS AND METHODS, row 5  ===============================  data  =>  dataset  4. MATERIALS AND METHODS, row 8  ===============================  dynamic  =>  the dynamic  5. RESULTS, row 2  =================  You write about figures 2 and 3. Describe also what Figure 5 shows!  Figure 2, 3, 5. IMPORTANT!  ==========================  You need to help the reader!  The arrows should be numbered and the respective artifact at each arrow  should be explained in the text.  FIGURE 2, figure text row 3  ===========================  vessel clarity, and  =>  vessel clarity than the  FIGURE 6, figure text row 2  ===========================  fast convergence over conventional  =>  faster convergence than conventional  6. DISCUSSION, row 9  ====================  XD-GRASP  =>  GRASP and XD-GRASP  6. DISCUSSION, row 13-15  ========================  allows up to 5x times and 3x time improvement in the reconstruction time compared to XD-GRASP and GRASP reconstructions respectively for Dataset-2 at AF=8.  =>  provides up to 5 and 3 times faster reconstruction time compared to XD-GRASP and GRASP reconstructions  for dataset 2 at AF=8, respectively. | Thanks for highlighting typo and grammatical mistakes. Now we have updated the draft and made all necessary corrections in the revised manuscript. |
|  | 6. DISCUSSION  =============  Are there any limitations with the new suggested method? | Thanks for the suggestion. We have added some future work in discussion section of the revised manuscript. |
|  | Figure 7  ========  This figure must also be referenced in the text!` | Thanks for the suggestion. Now we have discussed Figure 7 in the discussion section of the revised manuscript. |
|  | **Reviewer 2** | |
|  | **Major Comments** | |
| 1 | How MFISTA-VA is computationally better than the soft thresholding used in 1, 2, 6 and 9? | Thanks for highlighting the concern. Conventional L+S uses ISTA to obtain an optimal solution. In this work, we used MFISTA-VA to solve the L+S optimization problem which improves the convergence of Conventional L+S reconstruction. MFISTA-VA uses variable step size during iterative CS reconstruction which increases convergence of MFISTA-VA compared to FISTA and ISTA which use constant step size.  The revised manuscript contains a sub section 2.1.1 with details about MFISTA-VA. We have also added details about advantages of MFISTA-VA in the revised manuscript. |
| 2 | Can you explain the difference between this article? Shahzadi, Iram, et al. "Golden-angle radial sparse parallel MR image reconstruction using SC-GROG followed by iterative soft thresholding." Applied Magnetic Resonance 50 (2019): 977-988. | Thanks for highlighting the concern. The authors in [Ref. # a] integrate SC-GROG gridding followed by iterative soft thresholding (ISTA) reconstruction for radial DCE MRI which aims to improve reconstruction time only. While in our proposed method, we integrated SC-GROG followed M-FISTA-VA in L+S Reconstruction with multiple sparsity constraints. Major benefits of our proposed work are (i) improvement in convergence of conventional L+S reconstruction, (ii) improvement in reconstruction time of Golden angle radial DCE MRI and (iii) improvement in dynamic contrast of reconstructed DCE MR images.  [a]. Golden-angle radial sparse parallel MR image reconstruction using SC-GROG followed by iterative soft thresholding." *Applied Magnetic Resonance* 50 (2019): 977-988. |
| 3 | It seems your group has done quite a lot of work on GROG, GRASP and Compresses Sensing based reconstructions, it will be better if you can provide a comparison of results with this article as well. | Thanks for the concern. GRASP and XG-GRASP are contemporary techniques for accelerated free breathing radial DCE-MRI. We have compared our proposed method with these two techniques for fair comparison. Anyhow, we have added discussion of the related work done by our research group in the introduction section of the revised manuscript. |
| 4 | Section 2.2 contains a lot of information from the original paper by Ricardo Otazo about L+S Decomposition Model. It is better to reduce this section to show how MFISTA-VA is applied for L+S Model. | Thanks for highlighting the concern. Now we have added sub section 2.1.1 with more details about MFISTA-VA in the revised manuscript. We have also reduced details of L+S reconstruction in the revised manuscript. |
| 5 | Which algorithm was used to estimate the sensitivity maps? | Thanks for the concern. We estimated coil sensitivity maps by using the Walsh method [1]. This information has been added in the section 3.  [1] Walsh, D. O., Gmitro, A. F., & Marcellin, M. W. (2000). Adaptive reconstruction of phased array MR imagery. Magnetic Resonance in Medicine: An Official Journal of the International Society for Magnetic Resonance in Medicine, 43(5), 682-690. |
| 6 | Expression 3.2 there is no explanation how Temporal TV and Temporal FFT is applied on sparse data separately. | Thanks for highlighting the concern.  Temporal TV applies a finite-differences operator along the temporal dimension of the matrix. Similarly, temporal FFT converted the images series matrix from the y-t domain to the temporal-frequency (y-f) domain by applying an FFT operator along the temporal dimension.  We have added this information in the revised manuscript in the proposed method section (section 3). |
| 7 | “Table 4 shows that the peak DCE signal was degraded by 8.5% and 6.5% ….. ” Degraded in comparison of what? Did you use any reference image? How about adding a non-binned image for comparison? | Thanks for highlighting the concern. There was no reference image. We calculated signal intensity of all temporal frames in the selected region of interest (ROI) to see dynamic contrast variation among different temporal frame. This intensity was used to compare the dynamic contrast performance in all the reconstruction schemes. Tables 4 provides percentage improvement in dynamic contrast of proposed method compared to GRASP and XD-GRASP reconstruction.  The information has been updated in the revised manuscript. |
| 8 | In discussion section there is no mention of the reason why this SC GROG based method performs better than conventional methods. Which is very important for this section. | Thanks for the concern. SC-GROG is computationally efficient due to following factors, (i) it does not require some extra computations, e.g., density compensation function (DCF), convolution shapes and windows sizes and (ii) it does not require gridding and de-gridding (to maintain data consistency) during iterative CS reconstruction which make SC-GROG more efficient than the conventional NUFFT gridding approach. The following details have been updated in the discussion section of the revised manuscript. |
|  | **Minor Comments (Reviewer 2)** | |
| 1 | Introduction: Paragraph 1: “physiological movement (e.g., respiratory, and cardiac motion) introduces blurring artefacts”, these movements don’t only add up to the blurring artifacts, there are more artifacts linked with these movements. | Thanks for the concern. Now we have updated that sentence. |
| 2 | Introduction: Paragraph 3: “Temporal TV can suppress all the temporal variations”, how the spatial resolution is tackled? | Thanks for the concern. The following information has been updated in the discussion section of the revised manuscript:  Temporal TV is a kind of method to find the finite difference among the corresponding dimensions. In this work, we applied TV along the temporal dimension only. We use it to minimize the variation of pixels along the temporal dimension rather than between these pixels. Hence it will not affect the spatial resolution. |
| 3 | Introduction: Paragraph 5: Repetitive sentence. “To generate good quality DCE MR images, intensity variation due to contrast agent and the respiratory motion must be separated from each other before image reconstruction”. | Thanks for the correction. Now we have removed this repetitive sentence in the revised manuscript. |
| 4 | Theory Section: 2.1 Iterative Soft Thresholding Methods. It is good to talk about the thresholding methods, while on the other hand it is better to keep it precise to make it more readable. It is better to focus only on MFISTA-VA which is used in this paper. | Thanks for the suggestion. We have reduced L+S theory and have added a subsection to remain more focused on MFISTA-VA. |
| 5 | “Tables 4 provides the peak and mean DCE signal intensity values in the three reconstruction schemes for Dataset-1 and Dataset-2 respectively”, are you comparing the complete image or just the sparse images ? How it behaves if you just compare sparse images? | Thanks for highlighting the concern. We calculated dynamic signal intensity variation in the arterial region for the liver DCE-MRI dataset of all temporal frames to see dynamic contrast variation among different temporal frames. The peak and mean values of this intensity were used to compare the dynamic contrast performance in all the reconstruction schemes. We have added this information in the results section of the revised manuscript. |
| 6 | Figure 4 is a bit confusing. It is not clear if you are talking about 11 frames reconstruction, or 22 frames reconstruction in total or you are just showing results for these two particular frames. | Thanks for highlighting the concern. In free breathing DCE-MRI, Radial k-space data are continuously acquired using the Golden-angle scheme and sorted into time series by grouping an arbitrary number of consecutive spokes into temporal frames.  In the proposed method, firstly the acquired golden-angle radial DCE-MRI data are sorted into time frames. 1100 spokes with 512 readouts are acquired at a golden-angle increment, then by selecting 100 spokes in each time frame, generate 11-time frames, i.e., 1100/100=11 frames. The Nyquist sampling requirement for this case is 512 ∗ $\frac{\pi}{2}$ ≈ 803 projections, conforming to a simulated acceleration factor (AF) of 8.3, i.e., 803/100=8. Similarly, selecting 50 radial spokes in each temporal frame will generate 22 temporal frames (1100/50=11) which corresponds to simulated AF of 16 (803/50=16) [Ref # a].  We have added this information in section 4 of the revised manuscript.  [a] Feng, L., Grimm, R., Block, K. T., Chandarana, H., Kim, S., Xu, J., ... & Otazo, R. (2014). Golden‐angle radial sparse parallel MRI: combination of compressed sensing, parallel imaging, and golden‐angle radial sampling for fast and flexible dynamic volumetric MRI. Magnetic resonance in medicine, 72(3), 707-717. |
| 7 | There is not explanation of Figure 6 and Figure 7 in Results Section. And the plots are very blurry, and not suitable to be included in the paper in current form. | Thanks for highlighting the concern. Now we have added details about figures 6 and 7 in the results section of updated manuscript. We have provided the plots with better resolution in the revised manuscript. |
| 8 | Figure 6 and 7, needs improvement, as they both need all the methods mentioned for comparison in the results section. | Thanks for the concern. Now we have updated figures 6 and 7 in the revised manuscript. Our proposed method improves convergence of the conventional L+S reconstruction. We only compared convergence of L+S with the proposed method. It may not be feasible at this stage to compare the convergence properties of GRASP and XD-GRASP with our proposed method. |
|  | Formatting: There are quite a few grammatical errors, like  “NL-CG reconstruction which requires repeated NUFFT **ridding/degrading** to maintain”  “Where 𝑌𝑐 represents the under-sampled GROG-gridded Cartesian.”, complete this sentence. | Thanks for highlighting grammatical errors. We have updated the manuscript. |
|  | The placement of figures and tables needs to be corrected. Like Figure 4, comes before table 4, while you talk about it later in the text. Same for the Tables with pseudo codes for algorithms. | Thanks for the suggestion. We have updated the order of tables and figures in the revised manuscript. |
