# Supplementary material for: SC-GROG followed by L+S reconstruction with multiple sparsity constraints for accelerated Golden-angle-radial DCE-MRI
Source: PLoS One. 2025 Feb 14;20(2):e0318102. doi: 10.1371/journal.pone.0318102 (PMC11828349; doi:10.1371/journal.pone.0318102)
Supplement: Attachment — Submitted filename: Response to reviewers_minor.docx [file pone.0318102.s003.docx]

# Response to Review Comments

# (Manuscript Number:  PONE-D-24-06314R1)

We are thankful to the reviewers for their deep and thorough review. We have revised the manuscript in the light of their useful suggestions and comments. We hope that the revision has improved the paper to a level of their satisfaction. Number wise answers to the specific comments/suggestions/queries are as follows:

| **General comments** | | | |
| --- | --- | --- | --- |
| **Sr. No** | **Comments** | **Response** | |
|  | **Reviewer 1** | | |
| 1. | C1: The following text was below eq. (2.7) in the previous submission. Now it is missing.  "Here, the sparse matrix S models the grossly corrupted noise/outlier information which is assumed to be a very small fraction of the acquired MRI data. Lambda_T represents regularization parameters for S component." | Thanks for highlighting the concern. We have updated this information in the section 2.2 below eq. (2.7). | |
| 2 | IMPORTANT:  In Figure 5, I think the XD-GRASP image looks sharper and more detailed than the proposed work. Can you elaborate on that? | Thanks for highlighting this concern. Although XD-GRASP provides sharp response at some points in the reconstructed images, quantitative experimental results demonstrate that the proposed method consistently outperforms XD-GRASP, providing not only all the necessary information but also a significant improvement in reconstruction time.  We have added this information in Section 5 (Results) of the revised manuscript. | |
|  | **Reviewer 2** | | |
| 1. | Reviewer #2: For My previous Major Comment "Table 4 shows that the peak DCE signal was degraded by 8.5% and 6.5% ….. ” Degraded in comparison of what? Did you use any reference image? How about adding a non-binned image for comparison?"  If there is no reference data, the claim of improvement becomes less compelling. I strongly recommend comparing the results with non-binned data, as it would provide a clearer benchmark for evaluating dynamic contrast performance. Including such a comparison would significantly strengthen your conclusions, or alternatively, please provide a clear rationale for why non-binned data cannot be used. | | Thanks for highlighting the concern. Sorry for unclear description of Table 4 in the results section and table’s caption.  In continuously acquired golden angle radial MRI data, fully sampled acquisition (with respect to Nyquist criterion), is not performed [ref # a, b, c]. Therefore, the evaluation of the proposed method has been performed on the basis of quantitative metrics that do not require a fully sampled ground truth image as a reference. The results of the proposed method have been compared with the results of two conventional methods i.e. GRASP and XD-GRASP. Among these two methods, GRASP does not employ binning for reconstructing the dynamic contrast-enhanced data. So the comparison of the proposed method with GRASP corresponds to a comparison with non-binned data. We have added this information in the discussion section (Section 5) of the revised manuscript.  Table 4 shows comparison of peak and mean values of dynamic signal variation among different time frames in arterial region in the selected ROI of the proposed method compared to GRASP and XD-GRASP. The proposed method provides improved peak and mean dynamic contrast among these techniques due to additional sparsity constraint. We have updated this information in the table 4’s caption and also in the results section of the revised manuscript. Sorry for unclear description of the Table 4 in the results section and in table’s caption.   a. Feng L, Grimm R, Block KT, Chandarana H, Kim S, et al. Golden‐angle radial sparse parallel MRI: combination of compressed sensing, parallel imaging, and golden‐angle radial sampling for fast and flexible dynamic volumetric MRI. Magnetic resonance in medicine. 2014 Sep;72(3):707-17. b. Feng L. Golden‐angle radial MRI: basics, advances, and applications. Journal of Magnetic Resonance Imaging. 2022 Jul;56(1):45-62. c. Feng L, Axel L, Chandarana H, Block KT, Sodickson DK, et al. XD‐GRASP: golden‐angle radial MRI with reconstruction of extra motion‐state dimensions using compressed sensing. Magnetic resonance in medicine. 2016 Feb;75(2):775-88. |
